# Evaluation of measles and rubella integrated surveillance system in Apulia region, Italy, 3 years after its introduction

**DOI:** 10.1017/S0950268818000407

**Published:** 2018-03-13

**Authors:** I. A. Turiac, F. Fortunato, M. G. Cappelli, A. Morea, M. Chironna, Rosa Prato, D. Martinelli

**Affiliations:** 1 European Programme for Intervention Epidemiology Training (EPIET), European Centre for Disease Prevention and Control (ECDC), Stockholm, Sweden; 2 Department of Medical and Surgical Sciences, University of Foggia, Foggia, Italy; 3 Department of Biomedical Science and Human Oncology, Aldo Moro University of Bari, Bari, Italy

**Keywords:** Apulia, evaluation, Italy, measles, rubella, surveillance system

## Abstract

This study aimed at evaluating the integrated measles and rubella surveillance system (IMRSS) in Apulia region, Italy, from its introduction in 2013 to 30 June 2016. Measles and rubella case reports were extracted from IMRSS. We estimated system sensitivity at the level of case reporting, using the capture–recapture method for three data sources. Data quality was described as the completeness of variables and timeliness of notification as the median-time interval from symptoms onset to initial alert. The proportion of suspected cases with laboratory investigation, the rate of discarded cases and the origin of infection were also computed. A total of 127 measles and four rubella suspected cases were reported to IMRSS and 82 were laboratory confirmed. Focusing our analysis on measles, IMRSS sensitivity was 82% (95% CI: 75–87). Completeness was >98% for mandatory variables and 57% for ‘genotyping’. The median-time interval from symptoms onset to initial alert was 4.5 days, with a timeliness of notification of 33% (41 cases reported ⩽48 h). The proportion of laboratory investigation was 87%. The rate of discarded cases was 0.1 per 100 000 inhabitants per year. The origin of infection was identified for 85% of cases. It is concluded that IMRSS provides good quality data and has good sensitivity; still efforts should be made to improve the completeness of laboratory-related variables, timeliness and to increase the rate of discarded cases.

Measles is one of the highest contagious viral diseases, causing deaths mostly among young children even though a safe and cost-effective vaccine is available since 1963. By contrast to measles, rubella, also known as ‘German Measles’, is generally a mild disease for children but can have serious consequences for pregnant women; the vaccine against it is available since 1969. Humans are the only natural host for sustaining measles and rubella virus transmission, which makes global eradication of these diseases feasible [1–3].

The WHO Global Vaccine Action Plan for 2011–2020 has established the target of measles and rubella elimination in at least five WHO Regions by 2020 and the Member States in all six Regions have established goals to eliminate measles by 2020 or before [3].

Historically, measles has been a notifiable disease in Italy since 1888 and rubella since 1970 [4]. As recommended by WHO Plan for measles and rubella elimination, an integrated measles and rubella surveillance system (IMRSS) was established in 2013 in Italy. Its objectives include detecting sporadic cases and outbreaks, monitoring trends in incidence and circulation of viral genotypes, understanding transmission of infection, supporting case management, measuring and documenting the progress towards elimination [5].

Within the quality improvement activities, periodic evaluation of the IMRSS is needed to ensure that the stated objectives are being accomplished. However, since its establishment, the IMRSS was never assessed.

The purpose of this study was to describe and assess key attributes of the IMRSS in Apulia, a large Italian Region, to ascertain the extent to which stated objectives are achieved and to make recommendations for improvements.

In Italy, measles vaccination was recommended by the Ministry of Health since 1979 and rubella vaccination was introduced in 1972 (only recommended for girls in pre-adolescent age). Since 1999, the national Immunisation Plan scheduled a two doses universal routine (both boys and girls) programme using MMR (measles–mumps–rubella) combined vaccines [6]. In the Apulia region, coverage for one dose among children <24 months progressively increased up to 92.7% in 2011.

The IMRSS is a passive case-based surveillance system that includes information on individual records related to measles and rubella events. In Apulia, it covers the entire Region, approximately 4 million inhabitants.

A suspected measles case is defined as fever and maculopapular rash and at least one of the following three symptoms: cough, coryza or conjunctivitis. A confirmed case is defined as positive measles virus identification by virus isolation, nucleic acid testing, antigen testing by DFA, or seroconversion and also meeting the clinical criteria [7]. An epidemiologically linked measles case is defined as a suspected case that has not been adequately tested by a laboratory and that was in contact with a laboratory-confirmed measles case 7–18 days before the onset of rash [8]. A suspected rubella case is defined as any person with sudden onset of generalised maculopapular rash and at least one of the following five symptoms: cervical adenopathy, sub-occipital adenopathy, post-auricular adenopathy, arthralgia and arthritis. A confirmed case is defined as positive at least one of the following three: isolation of rubella virus from a clinical specimen, detection of rubella virus nucleic acid in a clinical specimen, or rubella virus-specific antibody response (IgG) in serum or saliva. An epidemiologically linked rubella case is defined as a suspected case that has not been adequately tested by a laboratory and that was in contact with a laboratory-confirmed rubella case 12–23 days prior to the onset of the disease [8].

The national law [9] makes it a statutory requirement that suspected cases of measles and rubella be reported, sending a paper form to the relevant Local Public Health Authorities (LPHA) by the diagnosing clinician/physician within 12 h. In the Apulia region, the LPHA has to report cases to the Regional Observatory for Epidemiology (ROE) within 24 h by fax or e-mail and has to initiate an epidemiological investigation, to collect samples within 48 h of receipt of the report, to conduct case finding and contact tracing. After an authentication procedure, a designated authorised physician from ROE, enters, by manual input, the received data into the web platform of the IMRSS and sends them to the National Institute of Health (NIH) within 24 h. The NIH reports to the Italian Ministry of Health, European Centre for Disease Control (ECDC) and WHO.

At the regional level, data are periodically analysed in an aggregated format, in order to evaluate the temporal trend of diseases and surveillance data are published in biannual reports on the ROE website. In addition, the NIH requests from the ROE, on annual basis, a report on the updated measles and rubella elimination status to be transmitted to the WHO.

Our evaluation design took into consideration guidelines, indicators and targets provided by WHO surveillance performance protocol [8] and ECDC [10]. The selected attributes for the evaluation of the Apulian IMRSS were sensitivity and completeness of reporting, timeliness of notification, the proportion of suspected cases with laboratory investigation, the rate of discarded cases and origin of infection.

Data on suspected measles and rubella cases reported to the IMRSS between 1 January 2013 and 30 June 2016 were extracted. Reporting activity describes the epidemiology of cases in Apulia region for the abovementioned period. Categorical variables were summarised as counts and proportions and continuous variables presented using appropriate measures of central tendency and variation.

All statistical analyses were performed using Stata 12 (Stata Corp. College Station, USA) and Excel. The annual notification rate was calculated using Apulian mid-year population data from the Italian Bureau of Statistics and the following age categories 0–4, 5–14, 15–39 and 40–60 years.

We assessed the IMRSS *sensitivity of reporting*, which refers to the number of cases reported by the surveillance system, divided by the number of cases reported in the Apulian population during the study period. The capture–recapture method, proposed in the article of Sabine C. de Greeff [11], was used. In the Apulia region, two other external data sources are available for monitoring measles and rubella: national notifiable diseases surveillance system (NNDSS) and hospital discharge registry (HDR).

The NNDSS is a passive, case-based, compulsory comprehensive surveillance system. The notification database contains a unique patient number, date of birth, gender, first and last name, postal code, municipality, date of notification, date of first symptoms and date of diagnosis. The physicians are obliged to report the clinical suspected measles or rubella cases to the LPHA within 48 h and the last has to notify the cases to the ROE within 30 days. The ROE has to notify the Ministry of Health and the Regional Public Health Authority on monthly basis.

The Hospital Discharge Registry contains information about each patient discharged from public and private hospitals and includes data related to both clinical and organisational aspects of hospitalisation. Records include demographic information, dates of admission and discharge, diagnoses (one main and up to five secondary diagnoses) and therapeutic procedures performed during the hospitalisation, type of admission and in-hospital mortality. Clinical information is coded using the International Classification of Diseases, Ninth Revision, Clinical Modification (ICD9-CM).

Both notifications and hospital discharge records lack information on laboratory-confirmed cases.

We assumed that the Apulia region had a closed population in the considered years (0.65% increase, data on native- and foreign-born from Italian Bureau of Statistics; first assumption of the capture–recapture analysis). To ensure that each individual had the same chance of being included in all three sources (second assumption), the analysis included all reported cases (suspected or confirmed by lab or epi-linkage). For the capture–recapture analysis involving three or more sources, the independence assumption (third assumption) is not crucial because interaction terms can be incorporated into regression models to adjust for source dependencies [12]. The homogeneity of capture (fourth assumption) was directly fulfilled by the linkage of records between the three sources by using a unique ID number.

The three-source analysis was performed by fitting eight log-linear models using STATA's user-written program ‘recap’ module, providing standard three-source capture–recapture analyses without covariates [13]. The estimated total number of cases, which included the number of cases not registered in any of the three sources and the Confidence Interval were computed according to a goodness-of-fit-based method proposed by Regal and Hook [14]. The choice of the final model was based on the Akaike information criterion (AIC), Bayesian information criterion (BIC) and the *P*-value (*P* < 0.05) [15, 16]. The log-linear model with the lowest AIC, BIC and *P*-value (*P* < 0.05) was selected as the most valid model.

*Completeness* was assessed by determining the percentage of case records with recorded data on each chosen variable: *id number*, *date of reporting*, *patient's birth date*, *place of residence*, *sex*, *date of onset*, *vaccination status*, *last date of vaccination*, *number of vaccine doses if vaccinated*, *date of sampling*, *laboratory results of serologic tests (IgM)*, *genotyping*, *presence of pregnancy*, *contact with a confirmed case*, *contact with a pregnant woman*, *type of case (imported*, *autochthonous related with an autochthonous case*, *autochthonous related with an imported case*, *autochthonous with unknown source)*, *outcome of infection*, *outbreak* and *travel history*.

*Timeliness of notification* was assessed by calculating the time that elapsed from onset of symptoms to initial alert, expressed as the percentage of case-based reports to IMRSS submitted within 48 h of rash onset.

The *proportion of suspected cases with laboratory investigation* (for IgM and/or PCR) was calculated, yearly and for the whole period, as the proportion of suspected cases with adequate specimens collected and tested at the Apulia Regional Reference Laboratory for Measles and Rubella (excluding from the denominator any suspected cases not tested by a laboratory and (a) confirmed by epidemiological linkage, or (b) discarded as non-measles/non-rubella by epidemiological linkage to a laboratory-confirmed case of another communicable disease or epidemiological linkage to a measles or rubella IgM negative case).

The *rate of discarded cases* was calculated as the rate of suspected cases investigated and discarded as non-measles or non-rubella cases using laboratory testing in the Regional Reference Laboratory and/or epidemiological linkage to another confirmed disease.

We also calculated the proportion of suspected cases for which the *origin of infection* (e.g. imported, import-related, autochthonous related to an autochthonous case or autochthonous with unknown source) has been identified. Moreover, we described the relations between the laboratory identified genotypes and the origin of travel-related cases. A travel-related case was defined as a case that met the criterion of the history of travel to areas in which the virus is known to be circulating in the 7–18 days (for measles) or 12–23 days (for rubella) before the onset of rash/disease [8].

The WHO fixed-target for the assessment of timeliness of notification in ⩽48 h of rash onset, the rate of laboratory investigation and origin of infection indicators are ⩾80% and for the rate of discarded cases is at least two discarded cases per 100 000 population.

The study was approved by the Institutional Review Board at the Apulian Regional Observatory for Epidemiology (PROT:35/OER/2017, 23 March 2017) and conducted according to the principles expressed in the Declaration of Helsinki. Informed consent was not obtained from participants because both surveillance and hospitalisation data were provided and analysed anonymously.

During 1 January 2013 to 30 June 2016, 127 measles and four rubella suspected cases were reported to the IMRSS. Due to the low number of rubella cases, we focused our analysis on measles.

The annual notification rate for measles per 100 000 inhabitants was 0.8 in 2013, 1.8 in 2014, 0.3 in 2015 and 0.1 until 30 June 2016. The largest number of cases (*n* = 76) was reported in 2014, mainly due to an epidemic occurred in Brindisi province with 33 cases (27 laboratory confirmed) that was secondary to an outbreak occurred on a ship cruising the western Mediterranean Sea [17]. The yearly and monthly distribution of the 82 laboratory-confirmed measles cases is presented in Figure 1a. For the whole period, the median age of the 82 laboratory-confirmed measles cases was 25 years (range: 1–60 years, the most affected age group: 15–39 years with 54 cases – Fig. 1b); 51% males.

**Fig. 1.**
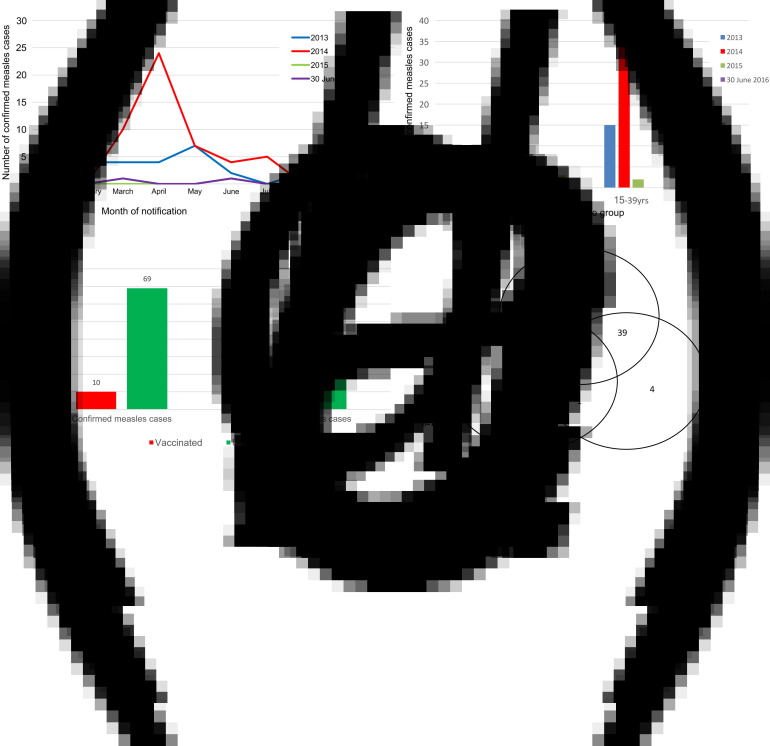
Evaluation features of the IMRSS in the Apulia region, Italy, 1 January 2013–30 June 2016. (a) Number of confirmed measles cases reported to the IMRSS by month of the report; (b) Age-specific distribution of measles confirmed cases per year; (c) Vaccination status of confirmed and suspected measles cases and (d) Venn diagram on the distribution of measles cases in three date sources without correction for false-positives.

The IMRSS also records the last date of vaccination and the number of vaccine doses received; sixty-nine (84%) confirmed and 35 (81%) suspected measles cases with a known vaccination status were unvaccinated. The vaccination status comparison among confirmed and suspected measles cases is presented in Figure 1c.

For computing the system *sensitivity of reporting*, 143 suspected cases had been recorded in at least one of the three sources. Of these, 52 were identified in all three sources, 39 were matches between source IMRSS and NNDSS, 11 were matches between source IMRSS and HDR and one was matches between source NNDSS and HDR (Fig. 1d).

The log-linear model with the lowest *P*-value (0.01), AIC (5.26) and BIC (5.37) included an interaction between NNDSS and HDR sources and provided an estimate of 154 total suspected cases. The overall sensitivity was estimated at 93% (95% CI 85–98). IMRSS sensitivity was 82% (95% CI 75–87). The sensitivity of NNDSS was 62% (95% CI 57–66) higher than that of HDR 49% (95% CI 44–51). As estimated by the model 7% cases were not identified by any of three sources.

*Completeness* was higher for the mandatory variables of the notification form and ranged between 98% (95% CI 94–99.8) for ‘date of onset’ and ‘vaccination status’ to 100% (95% CI 97–100) for ‘date of reporting’, ‘date of birth’, ‘place of residence’, ‘sex’, ‘outcome’, ‘outbreak’ and ‘travel history’. The incomplete variables were in the area of laboratory diagnosis, with 57% (95% CI 48–66) for ‘genotyping’, 67% (95% CI 58–75) for ‘laboratory results of serologic test’ and 76% (95% CI 68–83) for ‘sampling date’.

*The timeliness of notification* was computed for 124 cases. For two cases, the date of symptoms onset was missing and one case had chronological inconsistency (the date of initial alert was previous the rash onset date). The median length of time from the rash onset to initial alert was 4.5 days (range 0–54); the timeliness of notification was 33% (41/124) of cases reported within 48 h.

Noteworthy, the IMRSS does not record very important dates like the date of consultation, date of investigation, diagnosis date or date of test results, which could be helpful in allowing the assessment of timeliness of other steps of a surveillance system to accurately identify the patient, physician, diagnosis or laboratory delays and propose corrections.

Specimens collected for *laboratory investigation* were blood or serum for IgM testing and saliva (oral fluids) and/or urine for measles virus detection, isolation and genotyping (PCR). For the whole period, the number of measles reported cases with adequate specimens’ collection and available test results (IgM or PCR) was 97 out of 111 (excluding from the 127 total cases 16 confirmed by epidemiological linkage - Fig. 2) which yields a proportion of reported cases with laboratory investigation of 87%. The yearly proportion of laboratory investigation was 90% in 2013, 85% in 2014, 100% in 2015 and 80% for the first half of 2016.

**Fig. 2.**
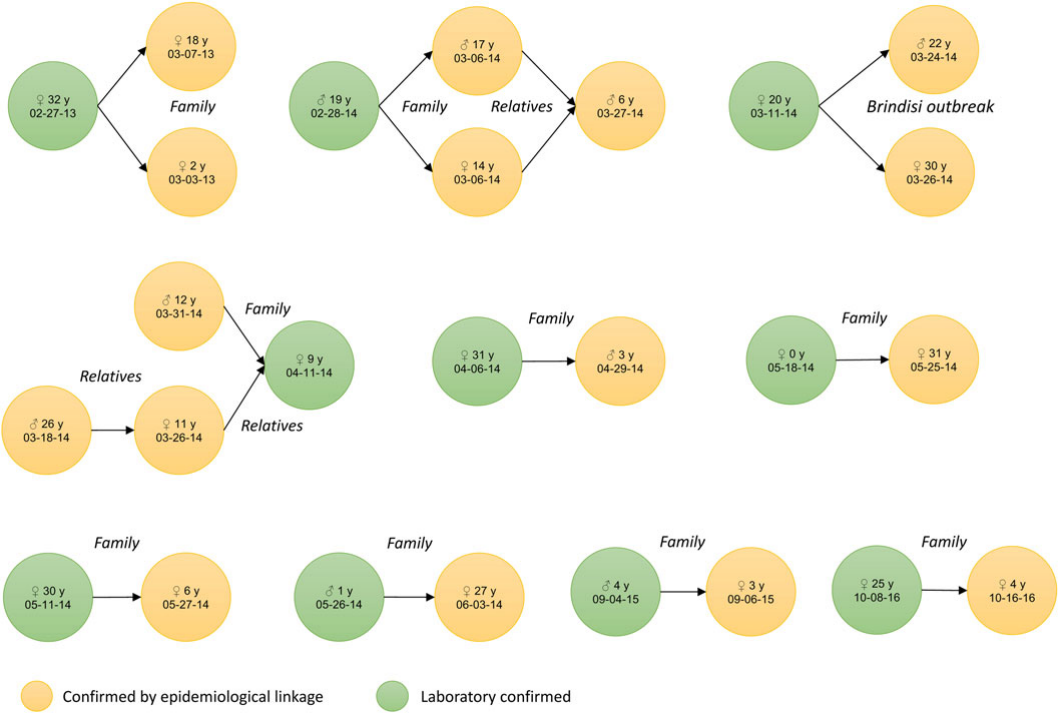
Measles cases confirmed by epidemiological linkage (*n* = 16), by sex, age and date of symptoms onset, Apulia region, Italy, 1 January 2013–30 June 2016.

Over the period, the average rate of discarded cases was 0.1 per 100 000 inhabitants per year.

A total of 108 (85%) suspected cases of measles were classified by the *origin of infection*. Fifty-five (43%) cases were identified as autochthonous with an unknown source, 38 (30%) cases as autochthonous related with an autochthonous case, eight (6.3%) cases as imported and seven (6%) as autochthonous related with an imported case. Nineteen (15%) cases were not classified in the given WHO classification.

The phylogenetic analysis performed for 47 confirmed cases identified genotypes B3 in 34 cases and D8 in 13 cases. Eight out of 10 cases who travelled abroad had the destination of their journey recorded in the IMRSS: four cases travelled to Spain (cruise ship passengers [17]), two cases travelled together to Thailand (onset 5–16 days after completion of travel), one to South Korea (onset 10 days after travel) and one to People's Republic of China (onset 5 days after completion of travel). The genotyping results were present in the database only for half of the cases (5/10) who travelled abroad. Genotype B3 was identified in the two travellers to Spain and in one to People's Republic of China; genotype D8 was found in two cases that went to Thailand.

The evaluation of the IMRSS, three and a half years after its implementation in the Apulia region of Italy, provided a good opportunity for the identification of strengths and areas that could be improved to accomplish the goal of measles and rubella elimination.

The IMRSS strengths are good sensitivity, high rate of laboratory investigation, the identified origin of infection for most of the cases and high level of completeness of the mandatory variables that demonstrates the high quality of the surveillance system and shows a strong engagement of the stakeholders who report to the system. The lower completeness of laboratory-related variables could be explained by the fact that those fields are not mandatory at the completion of the notification form that should supplement at a later stage. In addition, the IMRSS is a passive surveillance system, which relies mainly on the physicians, hospital or laboratory staff to take the initiative to report data and update information. The introduction of a reminder for the LPHAs from the ROE to update previously introduced information could improve both completeness and timeliness.

Since the IMRSS does not record the date of consultation, we cannot say for certain if the poor timeliness of notification, lower than the WHO target, could be due to patient delay or physician delay as well as to a low acceptability of notification obligation among some physicians, not completely aware of the necessity and importance of rapidly reporting, beginning investigations and implementing control measures, crucial in preventing secondary cases and outbreaks and disease elimination.

The lower than the WHO threshold rate of discarded cases could be explained by the attitude of the Apulian physicians that are notifying only the cases with clear symptoms or epidemiologically linked to another case or, sometimes, only laboratory-confirmed, despite the statutory requirement of reporting all suspected cases. Similar issues of biases in reporting were documented in many other countries [18], making the assessment of sensitivity and quality of the surveillance systems more difficult. In countries approaching measles/rubella elimination, a strategy to increase rates of discarded cases would be to shift to a more sensitive case definition (e.g. ‘rash-fever disease’). Since these symptoms are rather non-specific, laboratory confirmation is essential for differential diagnosis when the majority of clinically suspected cases are caused by other pathogens. Laboratories may also consider establishing diagnostic tests for identification of other aetiologies and rejection of suspected cases as non-measles and non-rubella [19].

Moreover, the low number of rubella cases reported in Apulia during the study period would be encouraging for rubella control, but the low rate of discarded cases raises the issues of clinicians missing potential cases. Given the higher efficacy of rubella vaccine and the lower *R*_0_ for rubella compared with measles, rubella eradication may be easier to achieve than measles eradication [20]. In Italy, from 2005 to 2015, a total of 8450 rubella cases were reported to the national surveillance system, of which 6183 were reported in 2008 [21]. Since 2013, an average of 38 cases has been reported each year, reflecting changes in rubella epidemiology and outbreak patterns [22]. However, recent serological survey data for rubella in the Apulian population showed that a pocket of 13.3% persons aged 17–65 years still lack immunity against rubella [23].

Our findings are subject to some limitations. In the sensitivity analysis, despite we made the four capture–recapture assumptions, we could not ensure a homogeneity of inclusion probabilities of all reported cases in the three sources. The nature of disease does not require necessarily hospitalisation, even though in the measles elimination phase the likelihood to be hospitalised is high and in Italy almost half of all measles cases reported in 2016 (46%, 388/844) required hospitalisation [22]. Moreover, we could not assess the outbreak detection capability of the system but we encourage that future evaluations include this to support the detection of space–time clusters using all available linked data.

Another limitation of our study is related to the assessment of the rate of laboratory investigation and the WHO requirement that samples must be tested in a proficient laboratory. In the Apulia region, samples were tested at the Regional Reference Laboratory that is undergoing an accreditation procedure to become one. In Italy, the only proficient laboratory belongs to the National Public Health Institute (ISS) in Rome.

Our study has identified a number of key recommendations like the introduction of new fields for several important dates mentioned above that will allow in the future the identification of timeliness of different surveillance steps and help in its enhancement. In addition, a greater attention should be given to chronological consistency of variables comprising dates, respecting the information flow of the notification procedure. A major recommendation is raising awareness of the importance of timeliness and data completeness among the healthcare workers of the Apulian LPHAs that will improve the quality of data and could allow a better assessment of timeliness. Moreover, the introduction of a reminder (phone call/e-mail) to the LPHA involved, to update the prior sent information, could increase both timeliness and data completeness.

In conclusion, our study shows that the Apulian IMRSS has good sensitivity, provides quality data and meets targets according to the WHO plan for eliminating measles and rubella [8]. Further sustained efforts should be made to improve timeliness, completeness of laboratory-related variables and rate of discarded cases of the IMRSS by raising awareness amongst Apulian physicians and the Local Public Health Authorities of its importance in achieving the elimination goals.
